# Supplementary material for: Rapid turnover of life-cycle-related genes in the brown algae
Source: Genome Biol. 2019 Feb 14;20:35. doi: 10.1186/s13059-019-1630-6 (PMC6374913; doi:10.1186/s13059-019-1630-6)
Supplement: Supplementary file 3 — Protein alignments, corresponding Gblocks html file and CDS sequences for the lineage conserved gametophyte-biased genes. (ZIP 2109 kb) [file 13059_2019_1630_MOESM3_ESM.zip › Con_GA_alignments/Ec-01_008910.aa_ali.fasta-gb.htm]

Ec-01\_008910.aa.fasta.trans.aa\_ali.fasta


## Gblocks 0.91b Results

Processed file: **Ec-01\_008910.aa.fasta.trans.aa\_ali.fasta**  
Number of sequences: **2**  
Alignment assumed to be: **Protein**  
New number of positions: **628** (selected positions are underlined in blue)

```
                         10        20        30        40        50        60
                 =========+=========+=========+=========+=========+=========+
Ec-01_008910     ---MPLTAEYTWAESETIIQVQVPLKGQSASKLDVFATECYLKVSFPPYLLEVDLDGYID
MSTRG.15789.1    DIKMPLIPEFTWVESKTTLQLRVPLKGHSKSNLDVFATECYLKVSFPPYLLEVDLDGCID
                    #########################################################


                         70        80        90       100       110       120
                 =========+=========+=========+=========+=========+=========+
Ec-01_008910     SMQPKARVENGTLMIKAKKKEGSARLWGSLGQRVSRKCDPKVKARREESIKEKHKLEDEL
MSTRG.15789.1    SVQPRARVDKGTLIIKAKKKEGSITLWGSFGRLADRKSSAESKNRREESIKEKFKQEQQL
                 ############################################################


                        130       140       150       160       170       180
                 =========+=========+=========+=========+=========+=========+
Ec-01_008910     KERVSLRRHQDERLALRSQMSVEEDERRRIDDIKAEEKEKAEAEVYRAFAQLAAKQNRGD
MSTRG.15789.1    KERVSLRKHQDERLALRSQMNLEEDERRRIEDIKAKEKEKAEAEVYRTFAELAAKQSRED
                 ############################################################


                        190       200       210       220       230       240
                 =========+=========+=========+=========+=========+=========+
Ec-01_008910     SAG-------TEAQPKEARSQGRHQQDKFTLPPSSTCSPRDRLHTIVEGVSDEHSPTPEI
MSTRG.15789.1    SQSTGKVPAPVNAQPKDMGSQRPNQQGRPTQPESRVCAPRHHLDTIDEGISEERSPNPTA
                 #           ################################################


                        250       260       270       280       290       300
                 =========+=========+=========+=========+=========+=========+
Ec-01_008910     MAPAYTAATKVDAGNSIWQEETATIKDGDRGYQEADGESERSGSTSATAESSWSSVEFTQ
MSTRG.15789.1    IASGATAAAKVECESSVSEEGADGSHSGDEKNDILHRKPERLSGGSGSHDPDALNEQGKE
                 #############################                               


                        310       320       330       340       350       360
                 =========+=========+=========+=========+=========+=========+
Ec-01_008910     QD---EQEEQQGDKTDAQHSDEDETPVSYTPAPRTSVGVIPVGFTTRLFPTPLRESKVAD
MSTRG.15789.1    QGKRGDVDAAPLEISDAGDDDDDDTTVSFTPAPRTSTGVVEVGFTTRLFPTPLRESKVAD
                                #############################################


                        370       380       390       400       410       420
                 =========+=========+=========+=========+=========+=========+
Ec-01_008910     ESAWIMKNRRHLHKNHALVGRLPAAGSGSAGVGGGDKDIPESDPVWLKGKGDDLYRGGDF
MSTRG.15789.1    ESAWIMKNRRHLHKNKSLVGRLPAGQQGSGRGEELPLDISESDPVWLKGRGDDLYRGGDF
                 ############################################################


                        430       440       450       460       470       480
                 =========+=========+=========+=========+=========+=========+
Ec-01_008910     LGAVNAYTAALDADPRAAACLSNRAACYLRLSGCNSSSHDQ---RRRHASACVADCGAAL
MSTRG.15789.1    LAAVNAYTAALDADPQAAACLSNRAACYLQLGGAGNGSNGNNGSRHEYASACVADCGAAL
                 ######################################      ################


                        490       500       510       520       530       540
                 =========+=========+=========+=========+=========+=========+
Ec-01_008910     EILRALPETGPSQARALARRSLAYRELGHYQSSLEDCRAALLFSPGDAALLEEAAQGEPL
MSTRG.15789.1    EILQTLPETGPSQGRALVRRSLAYLELGQYQCSLEDCRAALLFNPGDAGILGEIARGEPL
                 ############################################################


                        550       560       570       580       590       600
                 =========+=========+=========+=========+=========+=========+
Ec-01_008910     AQAERCKKEGATRFAAGDVRGACELYTTALEAVPGFPPALSNRAACYLALGKPGECVRDC
MSTRG.15789.1    ARGERCKKEAATRFASGDIAGACEMYTLALEAVPSLPSALSNRAACYLALAKPRECVRDC
                 ############################################################


                        610       620       630       640       650       660
                 =========+=========+=========+=========+=========+=========+
Ec-01_008910     TAVLEMLSASDTDLLSLLCDDHDHNHQHPIPRAGSSPTESAAPVENEKEQERASPFPATA
MSTRG.15789.1    TAVLQMLSAPVSGGGTSVPSAGGSGCSQDTACTSAVQADGGSDVE-QRPSRCSTSGVASP
                 #########                                                ###


                        670       680       690       700       710       720
                 =========+=========+=========+=========+=========+=========+
Ec-01_008910     AADRTPASPPPPPPPPVGSLPPPGSEKRTKWVMTTVARRARANLQLDNVEGALRDFRTAN
MSTRG.15789.1    QPEVAPSSTPPLPPPPVGSMPPPGSEKRTRWVLATLLRRARANVQLADLEGALRDYRMAN
                 ############################################################


                        730       740
                 =========+=========+=========
Ec-01_008910     SICPGNEGVEEDIRELERRINEA------
MSTRG.15789.1    LVLPGDKSVEEDIRELELRASEMQRPLVG
                 ######################
```

```
Parameters used
Minimum Number Of Sequences For A Conserved Position: 2
Minimum Number Of Sequences For A Flanking Position: 2
Maximum Number Of Contiguous Nonconserved Positions: 8
Minimum Length Of A Block: 10
Allowed Gap Positions: None
Use Similarity Matrices: Yes
```

```
Flank positions of the 5 selected block(s)
Flanks: [4  181]  [193  269]  [316  458]  [465  609]  [658  742]  

New number of positions in Ec-01_008910.aa.fasta.trans.aa_ali.fasta-gb:  628  (83% of the original 749 positions)
```
